# Supplementary material for: Transfer learning of multicellular organization via single-cell and spatial transcriptomics
Source: PLoS Comput Biol. 2025 Apr 21;21(4):e1012991. doi: 10.1371/journal.pcbi.1012991 (PMC12061427; doi:10.1371/journal.pcbi.1012991)
Supplement: Attachment — Submitted filename: Response to Reviewers.pdf [file pcbi.1012991.s002.pdf]

Reply letter

Dear Editor,

We would like to thank you and the three referees for evaluating our manuscript and for providing the constructive suggestions. To address all the concerns from the reviewers, we have carefully and extensively revised the manuscript (The high-resolution versions of the figures can be downloaded from the links). The major revisions are as follows:

1. **Expanded Benchmarking Comparisons**: The scope of the benchmarking analysis has been expanded to include comparisons between iSORT and the methods suggested by the reviewers, including CytoSPACE, RCTD, CARD, Redeconve, and Celloc. These comparisons demonstrate the robustness and effectiveness of iSORT in reconstructing spatial organization.
2. **Toy Model Simulation for SOG Explanation**: A simulation experiment was designed using a toy model to better explain the unique characteristics of spatial-organizing genes (SOGs) and to clearly distinguish them from spatially variable genes (SVGs).
3. **Application of SpaRNA Velocity to Complex Tissue:** iSORT's SpaRNA velocity has been applied to a more complex mouse embryo dataset, demonstrating its ability to infer spatially resolved developmental trajectories in a highly heterogeneous tissue environment. Additionally, we explored SIRV in the Supplementary Information (SI).
4. **Noise Sensitivity Analysis**: A noise sensitivity experiment was conducted to validate iSORT's robustness under varying noise conditions. The results confirm iSORT's consistent performance in reconstructing spatial organization, even with increasing noise levels.
5. **Additional Experiments on Brain Reconstruction:** To further evaluate iSORT across different spatial transcriptomics technologies, we conducted two additional experiments. First, we reconstructed the spatial organization of the mouse half-brain

using **10X Visium and MERFISH**, assessing iSORT's ability to infer spatial positions from different spatial transcriptomics platforms. Second, we applied iSORT to reconstruct the **human middle temporal gyrus (MTG) region**, using **MERFISH and Smart-seq**, further testing its capability in integrating single-cell and spatial transcriptomics data. These experiments provide a more comprehensive evaluation of iSORT in handling diverse data sources.

In the revised version with this reply letter, we have addressed all reviewers' comments. Please find our detailed point-by-point responses in the following. All the revisions in the manuscript have been highlighted in red. As a result of these revisions, we believe that our manuscript has been significantly improved, and hope that this version will be acceptable for publication in *PLoS Computational Biology*.

Yours sincerely,

Jifan Shi, Wei Lin, Yan Yan, and Qing Nie

Response to Reviewer #1

Reviewer #1:

This manuscript presents a transfer learning framework for predicting the spatial positions of single cells based on scRNA-seq and spatial transcriptomics data. While the topic is interesting, the benchmark is conducted with low standards, with only mapping-based algorithms compared. In fact, many deconvolution-based algorithms have been proposed to solve the same questions, including RCTD, cell2location, DestVI, CARD, Redeconve, CytoSPACE, etc. Deconvolution-based algorithms have higher accuracy than mapping-based algorithms. Without demonstrating the advantages compared with currently available deconvolution-based algorithms, the current tool cannot be recommended for publication.

Reply 1.0. Thank you very much for your insightful comments. We sincerely appreciate your thoughtful feedback on the benchmarking analysis in our manuscript. In response, we would like to clarify the advantages of iSORT and explain the improvements made in the revised version. For clarity, our response is divided into three key points:

(1) iSORT predicts continuous single-cell spatial positions, while spot deconvolution methods estimate cell-type composition.

Deconvolution-based algorithms (such as RCTD, CARD and CytoSPACE) primarily focus on estimating the cellular composition within each **spot** or probabilistically assigning single-cell profiles to **spot-level** data. Although these methods are effective for cell-type deconvolution, they are inherently limited in accurately reconstructing the precise spatial positions of individual cells and their relative spatial relationships. In contrast, iSORT is specifically designed to directly predict the spatial positions of cells **in single-cell resolution with continuous spatial coordinates**, enabling the reconstruction of tissue spatial structures and cellular neighborhood environments. Therefore, our initial benchmarking focused on methods capable of single-cell spatial reconstruction.

We fully agree with the reviewer that comparing iSORT with deconvolution-based

methods is highly valuable. Spot deconvolution methods are powerful tools for integrating spatial transcriptomics and single-cell data and offer significant advantages in broadly understanding tissue organization and cellular composition. In response to the reviewer's comment, we have expanded our benchmarking analysis accordingly: In the revised *"Benchmarking iSORT in reconstructing the spatial organization of single cells"* section, we incorporated widely used deconvolution-based algorithms, including RCTD, CARD, Redeconve, and CytoSPACE, as well as the recently published Celloc, for comprehensive comparison. Due to the complex implementation of Cell2location and the high computational cost of DestVI, we prioritized methods that balance performance and feasibility.

Specifically, our updated results can be found on pages 4-5, line 117-145 of the main text. In this section, we expanded the scope of benchmarking by adding comparisons with five spot deconvolution methods (RCTD, CARD, Redeconve, CytoSPACE, and Celloc). The results demonstrate that iSORT can reconstruct the overall shape of ST slice ID151674 based on gene expression and excels in distinguishing layer structures (as shown in the Fig. 2(a) in the main text). In comparison, Redeconve and RCTD performed well among the spot deconvolution methods. Furthermore, we evaluated these methods using multiple quantitative metrics. The results show that iSORT outperformed the other ten methods across all metrics (see Fig. 2(b) in the main text and Fig. S1). These findings highlight that iSORT achieves competitive, and even superior performance in spatial reconstruction.

We have also included references to relevant studies in this section, as follows:

[40] Cable DM, Murray E, Zou LS, Goeva A, Macosko EZ, Chen F, et al. Robust decomposition of cell type mixtures in spatial transcriptomics. Nat Biotechnol. 2022;40(4):517–526.

[41] Ma Y, Zhou X. Spatially informed cell-type deconvolution for spatial transcriptomics. Nat Biotechnol. 2022;40(9):1349–1359.

[42] Zhou Z, Zhong Y, Zhang Z, Ren X. Spatial transcriptomics deconvolution at single-cell resolution using Redeconve. Nat Commun. 2023;14(1):7930.

[43] Vahid MR, Brown EL, Steen CB, Zhang W, Jeon HS, Kang M, et al. High-resolution alignment of single-cell and spatial transcriptomes with CytoSPACE. Nat Biotechnol. 2023;41(11):1543–1548.

<u>Please find the updated version of the paragraph below</u>:

(Line 117-145) The output of iSORT were compared with the ground truth (ST slice ID151674) as well as five other existing methods capable of predicting single-cell spatial positions: scSpace, Tangram, novoSpaRc, CeLEry, and CellTrek. Additionally, we performed a comparison with five spot deconvolution methods: RCTD, CARD, Redeconve, CytoSPACE, and Celloc, to provide a more comprehensive benchmarking analysis (Fig. 2(a)). It is worth noting that Tangram is designed for spot deconvolution, while novoSpaRc is designed for imputing undetected genes, but both methods use probabilistic models and can be used for position reconstruction. Detailed information on the other methods is shown in Supplementary Note 2. In the group of methods for spatial position reconstruction, iSORT, Tangram, novoSpaRc, and CeLEry could reconstruct the shape of ST slice ID151674 from gene expressions, while iSORT, scSpace, novoSpaRc, and CeLEry could distinguish different layers with clear boundaries. For the spot deconvolution methods, we selected the cell type with the highest proportion in each spot as the dominant cell type for that spot, providing a unified basis for comparison. Each method was able to roughly distinguish the different layers, particularly the white matter (WM) layer. Among the spot deconvolution methods, Redeconve and RCTD showed the clearest distinction of layers. In contrast, CARD did not predict the presence of Layer 6 cell types, as no spot in the CARD predictions had Layer 6 as the most abundant cell type. Celloc and CytoSPACE showed distinct results for layers other than the WM layer, but the overall layer differentiation was less pronounced compared to Redeconve and RCTD. Four different indicators were used to evaluate the performance of the eleven methods (Fig. 2(b)), i.e. the intra-layer similarity S_C, the normalized density distribution ρ_C, the aggregative volume index A_C, and the aggregative perimeter index P_C (Definitions and details are provided in Supplementary Note 5). Values of the indicators measure the performance of algorithms in reconstructing layer

structures. iSORT got an average S_C as 0.94, ρ_C as 0.84, A_C as 0.88, and P_C as 0.84, which were larger than the values obtained from the other ten methods (Fig. 2(b) and Fig. S1).

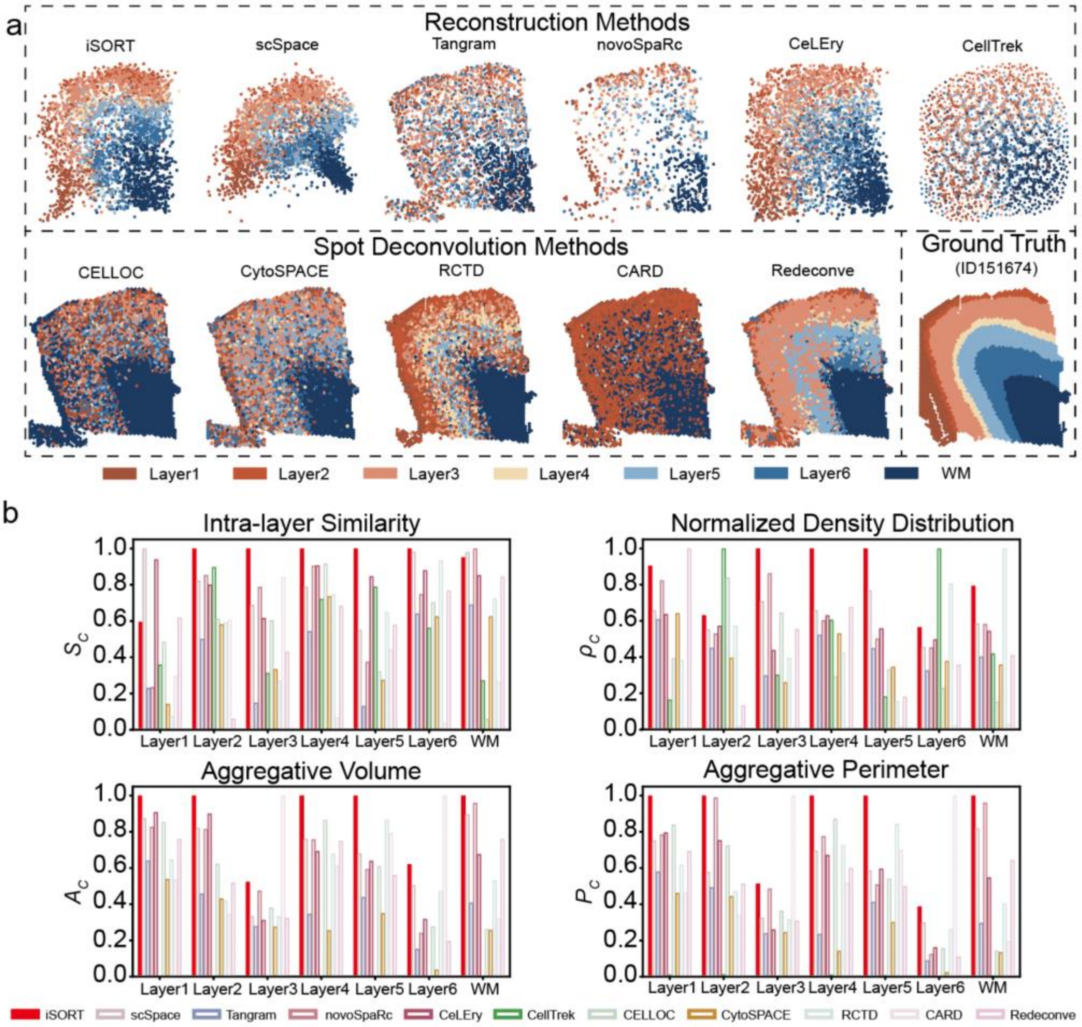

Figure 2(a) and (b). (a) Ground truth and spatial reconstruction results for ID151674 in DLPFC dataset by eleven algorithms: iSORT, scSpace, Tangram, novoSpaRc, CeLEry, CellTrek, RCTD, CARD, Redeconve, CytoSPACE, and Celloc, where Tangram, RCTD, CARD, Redeconve, CytoSPACE, and Celloc are designed for spot deconvolution, whereas novoSpaRc is designed for imputing the undetected genes. Different colors represent different cortical regions. (b) Bar charts to compare the performance of eleven algorithms based on four indicators: intra-layer similarity S_C, normalized density distribution ρ_C, aggregative volume index A_C and aggregative perimeter index P_C of each layer. The indicators of iSORT (the red filled bars) are above 0.875 in average, larger than the other ten algorithms.

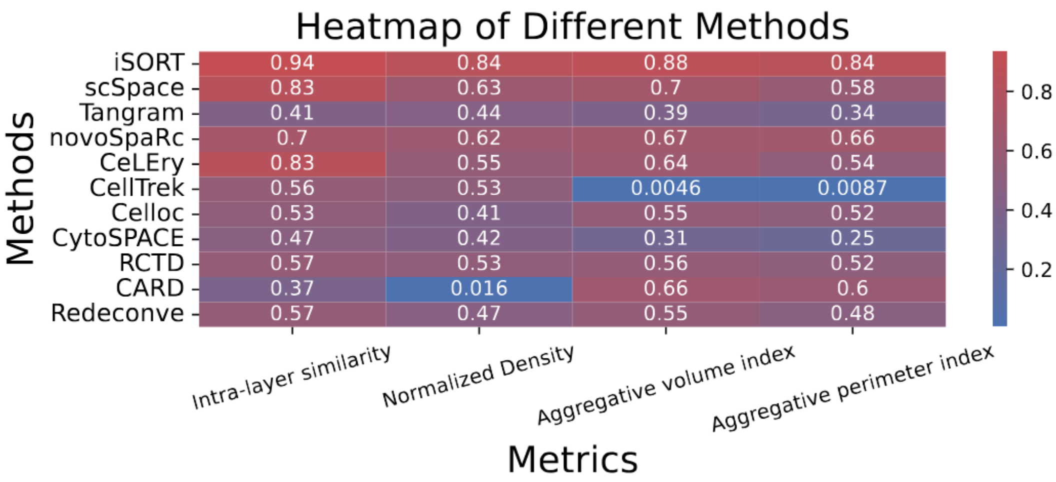

Supplementary Figure 1. Heatmap of four clustering metrics. Heatmap of four metrics (Mean of intra-layer similarity, mean of normalized density, mean of aggregative volume index and mean of aggregative perimeter index) of different methods (iSORT, scSpace, Tangram, novoSpaRc, CeLEry, CellTrek, RCTD, CARD, Redeconve, CytoSPACE, and Celloc). The redder the color, the better the clustering of the hierarchies. iSORT outperforms other methods in layering performance in an average sense.

(2) iSORT integrates multi-slice spatial data for improved reconstruction and state-specific gene discovery

Unlike most deconvolution-based methods, which aim to determine the cellular composition of spots within **individual slices**, iSORT is designed to **integrate data from one or multiple reference slices** during training, achieving a higher level of information fusion. This design has two significant advantages:

- Improved spatial reconstruction accuracy: Multi-slice integration enables iSORT to capture more comprehensive spatial information, leading to more precise reconstruction of tissue structures.
- Identification of spatial-organizing genes (SOGs): By using slices from different biological conditions (e.g., diseased vs. healthy) as references, iSORT can identify SOGs that reflect state-specific spatial differences. This capability is crucial for uncovering genes associated with spatial structural changes during

disease progression.

To emphasize this point, we added a comparison with spot deconvolution methods in the discussion section (line 484–495), highlighting the advantages of iSORT in integrating information from multiple slices.

<u>Please find the updated version of the paragraph below</u>:

(line 484–495) Traditional spot deconvolution methods typically assign single cells to spots or integrate data to infer the cell type proportions within spots. Although these methods achieve high accuracy, they are limited to analyzing a single ST slice and cannot utilize information from multiple slices. iSORT's multi-slice training approach overcomes this limitation. One aspect is that integrating data from multiple slices significantly improves the accuracy of spatial reconstruction. Another is that incorporating slices in different states enables iSORT effectively identify key genes that determine spatial structures. This capability makes iSORT uniquely advantageous in disease research — leveraging spatial information from multiple ST slices to identify SOGs, thus providing a method for detecting pathogenic genes. Besides the atherosclerosis described in this study, iSORT could offer a new perspective for revealing broader disease-related pathways.

(3) iSORT bridges spatial mapping with RNA velocity to reveal developmental and dynamic insights

As a mapping-based method, iSORT naturally allows for the integration of RNA velocity with spatial information. This unique feature enables the projection of dynamic cellular trajectories onto real spatial tissue structures, providing deeper insights into tissue development and spatial progression. In contrast, deconvolution-based methods generally lack this capability, limiting their ability to capture dynamic biological processes.

This point is also discussed in the discussion section (line 496–512), where we

elaborate on how iSORT leverages RNA velocity to integrate dynamic cellular trajectories with spatial information, which is not achievable with deconvolution-based methods.

<u>Please find the updated version of the paragraph below</u>:

(line 496–512) Moreover, iSORT's mapping-based approach allows it to incorporate the concept of RNA velocity to infer pseudo-growth trajectories on ST data, thereby capturing dynamic changes within tissues. By inferring SpaRNA velocity, iSORT not only reflects the organizational and differentiation processes within tissues, but also reveals spatiotemporal dynamics. More recently, SIRV was developed to infer RNA velocity in the physical space of ST by integrating scRNA-seq and ST data. Specifically, it utilizes splicing information from scRNA-seq to predict spliced and unspliced RNA levels in the ST data, enabling RNA velocity inference within the tissue structure. In contrast, we propose a quantity named SpaRNA velocity. Instead of relying on external sequencing references, SpaRNA velocity is computed solely from ST data and projected onto the ST slice, providing pseudo-growth trajectories that model how cells transition from their progenitors in space. Other methods of RNA velocity, such as scVelo, can also be appropriately modified for SpaRNA velocity inference. Besides, the inference of pseudo-time is another important aspect in assessing cell developmental status. Inference of SpaRNA velocity directly from ST data provides a promising framework for studying pseudo-time and could serve as a meaningful topic for future research.

We have summarized the above three key features in the Discussion section (line 480–483):

(480-483) Meanwhile, compared to traditional spot deconvolution methods, iSORT exhibits three advantages: (1) iSORT directly predicts the spatial positions of single cells in continuous space. (2) iSORT integrates information from multiple slices. (3) iSORT infers pseudo-growth trajectories using SpaRNA velocity.

We sincerely appreciate your constructive feedback, which has greatly improved the clarity and comprehensiveness of our manuscript. We highlight the features of iSORT and differences from deconvolution methods in the revised version, especially compared with ten methods.

Response to Reviewer #2

Reviewer #2:

Remarks to the Author:

In this study, Shi et al. present iSORT, an algorithm that combines single-cell data and spatial transcriptomics through a transfer learning approach to reconstruct spatial organization. The authors benchmark iSORT against state-of-the-art methods using datasets from the human dorsolateral prefrontal cortex (DLPFC), mouse embryo, and mouse brain, assessing its performance in spatial distribution reconstruction. iSORT effectively identifies spatial patterns at the single-cell level, pinpoints spatial-organizing genes (SOGs) that drive these patterns, and infers pseudo-growth trajectories using the concept of SpaRNA velocity. Overall, iSORT demonstrates accuracy and efficiency in mapping single-cell and spatial transcriptomics data. However, the authors should address several issues related to this methods.

Reply 2.0: Thank you for your insightful summary and positive comments on our work. We have thoroughly revised the manuscript to address all the concerns, and our detailed responses are presented below.

1. In this study, the most scRNA-seq input was derived from the gene expression of spatial transcriptomics (ST) data at each spot, with the spatial coordinates removed. Since Visium data does not achieve true single-cell resolution and SeqFish only detects thousands of genes, these cannot be considered genuine single-cell data. It is crucial to evaluate iSORT's performance on different ST platform paired with real single cell data to determine whether it can effectively address batch effects between single-cell and ST technologies, enabling the reconstruction of spatial patterns and facilitating downstream analyses.

Reply 2.1: We thank the reviewer for the insightful comments. We appreciate your observation regarding our use of scRNA-seq data derived from ST after removing spatial coordinates. You are correct that some of the scRNA-seq input in our study was

derived from ST. The primary reason for using this approach was to provide a **ground truth** for evaluating iSORT's performance. Since using real single-cell data would have made it difficult to directly assess the reconstruction results, this simplified simulation allowed us to more accurately test iSORT's ability to integrate and reconstruct spatial patterns. However, we acknowledge that this may not fully represent the real-world scenario, where scRNA-seq and ST data come from different samples and technologies.

In response to the reviewer's comment, we have added two new experiments to further evaluate iSORT's performance using real data from different technologies: we have added a subsection titled "*Reconstructing different brain regions with diverse technologies*" in the revised *"Benchmarking iSORT in reconstructing the spatial organization of single cells"* section (Line 171-195). To assess iSORT's ability to integrate data from different spatial transcriptomics technologies, we collected data from 10X Visium and MERFISH to conduct the mouse half-brain reconstruction experiment (Fig. S4). To assess iSORT's ability to integrate image-based ST data and scRNA-seq data, we collected MERFISH and Smart-seq data to conduct the human middle temporal gyrus (MTG) region reconstruction experiment (Fig. S5).

Currently, our manuscript includes four experiments directly assessing iSORT's spatial reconstruction ability using real data: 1) **Mouse brain spatial reconstruction**, where we combined Smart-seq and 10X Visium data to test iSORT's ability to integrate sequencing-based ST data with scRNA-seq data; 2) **Arterial tissue spatial mapping**, where we applied iSORT to integrate scRNA-seq and ST data from arterial tissue, demonstrating its applicability to different tissue types; 3) **Mouse brain hemisphere reconstruction**, where we combined 10X Visium and MERFISH data to evaluate iSORT's ability to integrate data from different platforms; and 4) **Human middle temporal gyrus region reconstruction**, where we integrated MERFISH and Smart-seq data to evaluate iSORT's ability to integrate image-based ST data with scRNA-seq data.

These experiments allowed us to evaluate iSORT's ability to reconstruct spatial

organization using real data from different tissues, providing valuable insights into its performance in practical scenarios. We believe these experiments help address concerns about the generalizability of iSORT and lay a solid foundation for understanding how iSORT handles real data. We hope this resolves your concerns, and we thank you for your feedback, which has helped clarify the scope of our current experiments and the robustness of iSORT in real-world applications.

Please find the updated version of the paragraph below:

(Line 171-195) Reconstructing different brain regions with diverse technologies. To further validate the performance of iSORT across different technical datasets, we designed two independent experiments. The first experiment aims to test the ability of iSORT to integrate sequencing-based and image-based technologies, particularly integrating MERFISH and 10X Visium technologies to reconstruct the spatial structure of the mouse half-brain. The 10X Visium dataset contains 5 slices, with each slice consisting of several thousand samples and covering approximately 30,000 genes, making it suitable for analyzing cortical region and layer structures. We selected one slice (ST8059048) for analysis. The MERFISH technology provides a brain atlas dataset, and we selected a region slice similar to the 10X Visium data (Fig. S4(a)), which contains over 100,000 cells and 550 genes, offering higher single-cell resolution and spatial coordinate information. By removing the coordinates from the 10X Visium data, we used the MERFISH slice as a reference to successfully perform spatial reconstruction of the 10X data. The results show that iSORT was able to clearly reconstruct the spatial structure of key brain regions, including multiple cortical layers, hippocampal regions, hypothalamus, and thalamus (Fig. S4(b)). The second experiment aims to test iSORT's ability to integrate image-based slices with single-cell data. We combined two human MTG datasets, one based on MERFISH and the other based on Smart-seq sequencing. Although the Smart-seq data lacks spatial coordinates for the cells, it includes anatomical information about brain subregions and manually

annotated cell types. In contrast, the MERFISH dataset provides single-cell resolution data with spatial coordinates and layer information. In this analysis, iSORT successfully reconstructed the spatial organization of the human MTG, accurately distinguishing cortical layers (L1–L6) and restoring their layered structure (Fig. S5).

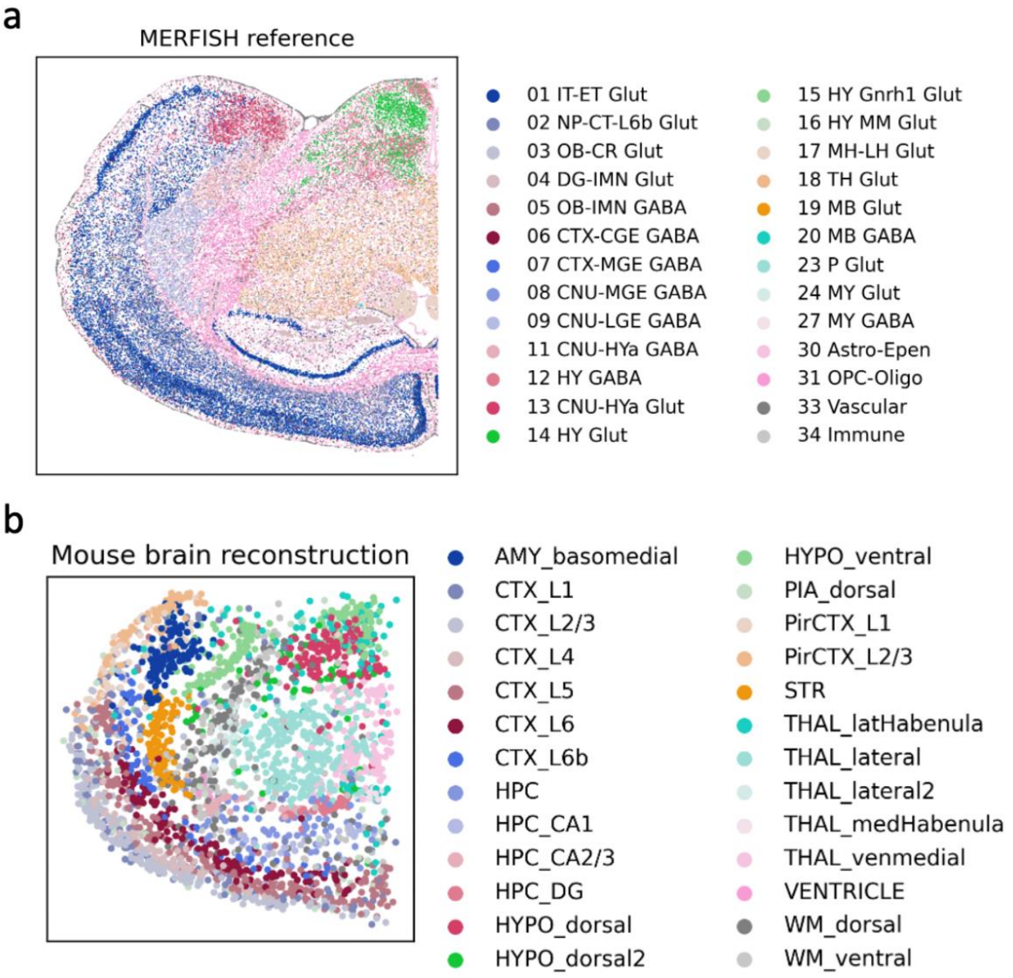

Supplementary Figure 4. Spatial reconstruction of mouse half-brain with iSORT. (a) MERFISH reference for mouse half-brain. (b) Spatial reconstruction of the mouse half-brain of 10X Visium, with MERFISH data as the reference.

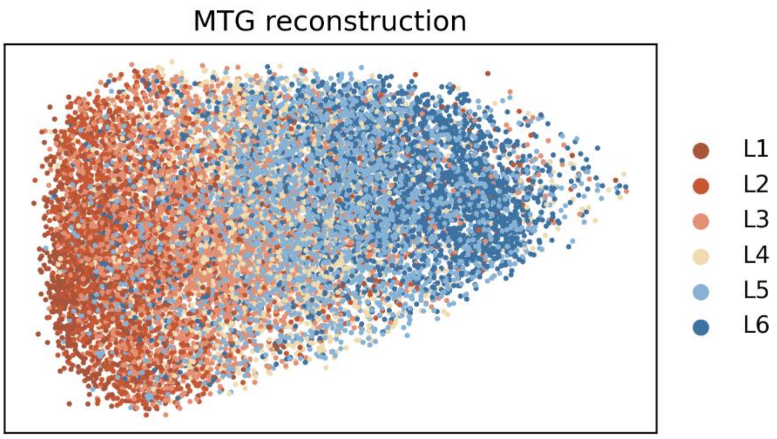

Supplementary Figure 5. Reconstructing the human middle temporal gyrus (MTG) with iSORT. Reconstruction of the human middle temporal gyrus (MTG) using scRNA-seq data with MERFISH as the reference. The result successfully delineates the MTG region and accurately differentiates between cortical layers L1, L2, L3, L4, L5, and L6.

2. The manuscript compares iSORT with other methods using simulated SC data. However, additional comparative analyses are necessary to demonstrate the robustness of iSORT's performance. The authors analyze single-cell data generated from spatial transcriptomics data, but this method may not accurately represent real-world situations where SC and ST data originate from different samples and different technologies. It could be beneficial for the authors to evaluate iSORT's performance by incorporating various noise levels into the SC data.

Reply 2.2: We thank the reviewer for the insightful feedback. We fully recognize the importance of evaluating the robustness of iSORT under more realistic conditions. In response, we have **incorporated a noise perturbation experiment** into the DLPFC dataset to rigorously assess iSORT's robustness under noisy conditions. Specifically, we introduced gene-specific Gaussian noise into the gene expression profiles of the simulated SC data. The noise had a mean of zero, and the variance was proportional to the intrinsic variability of each gene. Mathematically, the noise variance for gene i was modeled as the product of a scaling factor and the intrinsic variance of the gene, allowing us to precisely control the signal-to-noise ratio. To prevent biologically

implausible negative expression values, we imposed a lower bound of zero on the gene expression levels. This experimental design enabled us to flexibly adjust the noise intensity across the dataset and systematically evaluate iSORT's performance under varying noise conditions.

In response to the reviewer's comment, we have added a subsection titled "Noise sensitivity analysis for iSORT on DLPFC dataset" in the section " Robustness of iSORT in spatial reconstruction across different slices and noise conditions" (Line 259-271) to present the experimental results. In the Method section, we have also added a new section titled "Incorporating variable noise intensities into semi-simulated data" to detail the noise simulation experiment (Line 614-628). Our results show that even with increasing noise levels, iSORT was still able to successfully reconstruct the spatial organization of cells (Fig. S2(a)), although reconstruction performance declined as the noise level increased (3 times the intrinsic variability of the genes) (Figs. S2(b) and S3).

Once again, we sincerely thank you for your constructive suggestion, which has helped further validate and strengthen the reliability of iSORT.

Please find the updated version of the paragraph below:

(Line 259-271) Noise sensitivity analysis for iSORT on DLPFC dataset.

To assess the robustness of iSORT under varying noise conditions, we performed a noise sensitivity analysis on the semi-simulated scRNA-seq data from ST slice ID151674. Gaussian noise was added to the data at varying levels, with the noise intensity controlled by the parameter σ (details in Methods). We tested noise levels of $\sigma = 0.1, 0.5, 0.8, 1, 2, 3$, simulating increasing levels of noise. The results showed that iSORT performed well within the range of σ = 0 to 1, successfully reconstructing the spatial organization of the DLPFC tissue (Fig. S9(a)). However, when σ increased to 2, some performance metrics, such as intra-layer similarity S_C and the aggregative volume index A_C, dropped below 0.3 on average (Figs. S9(b) and S10). At $\sigma = 3$, the decline became more pronounced, with certain values falling below 0.1, indicating a significant decrease in

reconstruction accuracy. Despite this decline, iSORT preserved some spatial structure, indicating partial robustness under high noise levels.

(Line 614-628) Incorporating variable noise intensities into semi-simulated data.

To evaluate the robustness of iSORT under varying noise conditions, we incorporated Gaussian noise into the single-cell gene expression data. The noise was added at a gene-specific level, with the intensity controlled by a noise ratio parameter σ, which adjusts the proportion of noise relative to the intrinsic variability of each gene. This process simulates realistic noise levels in scRNA-seq data and assesses the impact of noise on iSORT's ability to reconstruct spatial patterns.

Let X_{ij} be the expression value of gene j in cell i, and $Var(X_j)$ the intrinsic variance of gene j across cells. The Gaussian noise for each gene is generated as:

$$\mathcal{N}_{ij} \sim \mathcal{N}(0, \sqrt{\sigma \cdot Var(X_j)})$$

where $\mathcal{N}_{ij}$ represents the noise added to X_{ij}. The noise ratio σ determines the noise intensity, with higher values corresponding to more noise. For instance, $\sigma = 0.1$ introduces 10% noise variance relative to the gene's intrinsic variance.

The noisy gene expression matrix X' is then computed as:

$$X'_{ij} = max(X_{ij} + \mathcal{N}_{ij}, 0)$$

ensuring non-negative values for the final expression data.

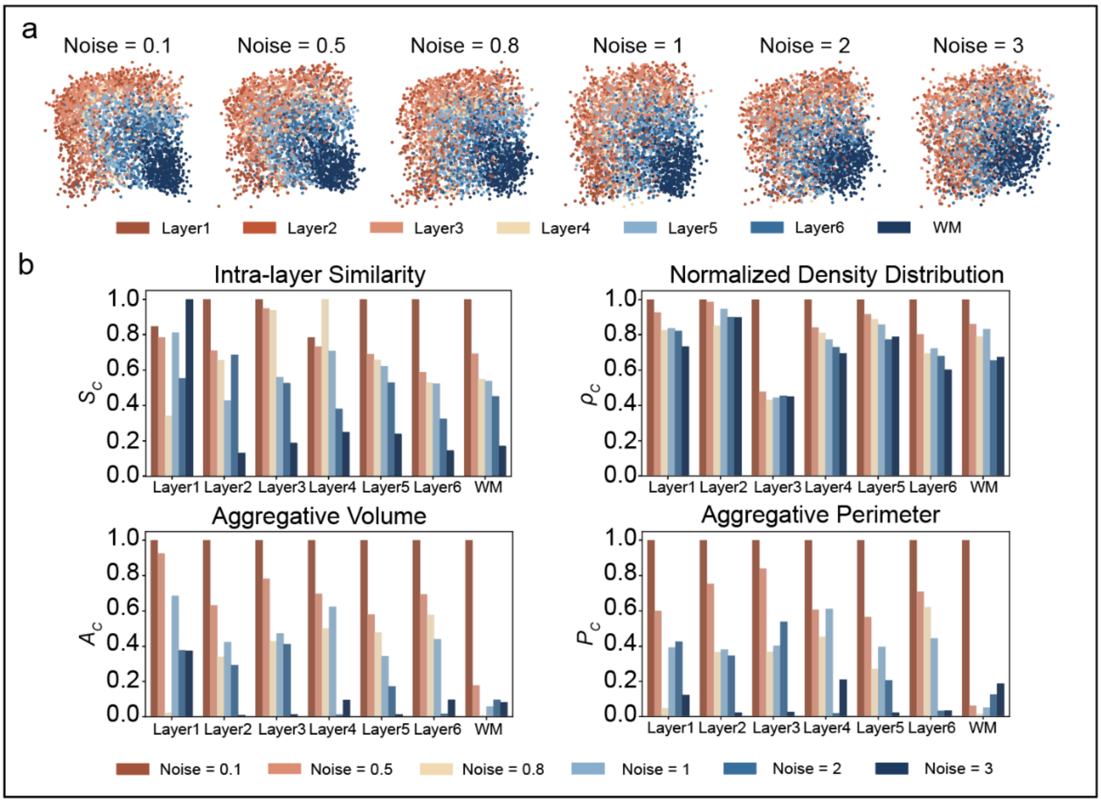

Supplementary Figure 9. Spatial organization reconstruction by iSORT under noise conditions in the DLPFC. (a) Spatial reconstruction results for ID151674 in the DLPFC dataset when noise is added to gene expression. (b) Bar charts to compare the reconstructed performance based on four indicators.

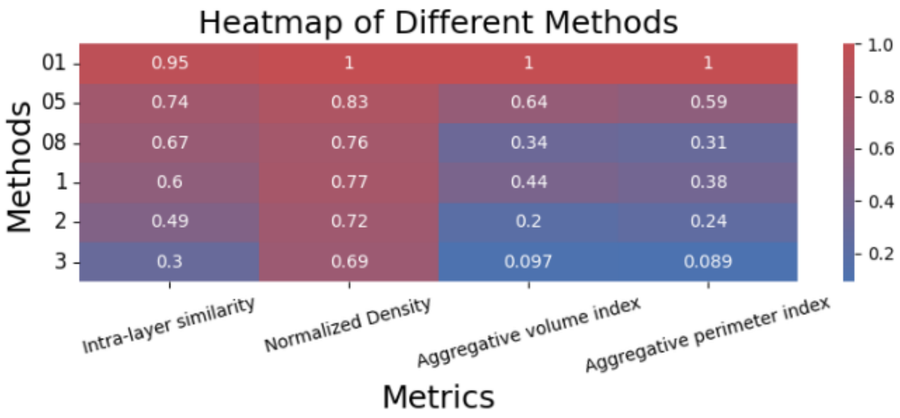

Supplementary Figure 10. Heatmap of four clustering metrics when noise is added in the DLPFC.

3. When the single-cell data and spatial transcriptomics data have different numbers of genes, how should genes be selected for integration, particularly when the ST data,

obtained from image-based technologies, includes only a few hundred genes? Can iSORT assist in identifying more spatially dominant genes from the scRNA-seq data, which contains a larger gene set?

Reply 2.3: We thank the reviewer for raising the issue about the integration of single-cell RNA-seq (scRNA-seq) and spatial transcriptomics (ST) data with different numbers of genes. In response to the comment, we have revised the manuscript to clarify the process of gene selection and have now provided a more detailed description as follows:

(1) Gene selection for integration

When integrating scRNA-seq and ST data with different numbers of genes, iSORT adopts a shared gene strategy during the spatial reconstruction phase. Specifically, for seq-based datasets, we first select the highly variable genes from both datasets and then extract the common subset as the feature set. For image-based datasets with fewer genes, we directly select the common gene set between the two datasets.

In response to this comment, we have expanded the description in lines 528–531, "Highly variable genes are selected for further studies," to "Next, highly variable genes are selected separately from the scRNA-seq and ST datasets. Then, we select the common subset of highly variable genes between the two datasets as the genes for subsequent tasks."

(2) Identification of spatially dominant genes

Regarding the identification of additional spatially dominant genes from scRNA-seq data, this is not performed during the initial integration of scRNA-seq and ST datasets or during the spatial prediction step, as only the shared gene subset is utilized at this stage. However, to expand the scope of gene exploration beyond the shared gene set, gene imputation techniques can be employed to infer the expression of missing genes, thereby incorporating non-shared genes into the analysis. With this expanded gene set, iSORT can be trained on a broader set of genes rather than being restricted to the initial

common subset. Following this, we can conduct a post-reconstruction analysis to determine SOGs from the expanded gene set, assess their spatial dominance, and select key genes for downstream spatial analysis. This is part of our future research efforts to further enhance iSORT's capability in integrating ST and single-cell data while enabling a more comprehensive identification of spatially dominant genes.

To clarify this point, we have added a discussion on identifying additional spatially dominant genes from scRNA-seq data in the main text (page 13-14, lines 464–474). We sincerely appreciate the reviewer's valuable suggestions, which have helped us better highlight the potential and flexibility of iSORT.

<u>Please find the updated version of the paragraph below</u>:

(Line 464–474) Second, although we employ a shared gene strategy during the training phase, we can first perform gene imputation on both datasets before training, thereby expanding the available gene set beyond the originally shared genes. With this expanded gene set, iSORT can be trained on a broader set of genes rather than being restricted to the initial common subset, allowing for a more comprehensive spatial reconstruction of single-cell organization. Subsequently, we can identify SOGs from this expanded set, assess their spatial dominance, and further select them as spatially dominant genes for downstream spatial analysis. This approach allows researchers to explore potential SOGs that were not initially included in the shared gene subset, thereby enhancing iSORT's utility in investigating the spatial roles of specific genes and improving the overall robustness of ST integration.

4. The authors claim that the SOGs identified by iSORT are more effective than the SVGs detected by Moran's I and SpatialDE based on gene knockout experiments. What are the key differences genes between the SOGs and SVGs? The authors should provide additional evidence, such as gene expression patterns for the SOGs, and discuss the

biological significance of these genes and their relationship to tissue structure and domains.

Reply 2.4: We thank the reviewer for valuable and insightful comments. We are pleased to have the opportunity to clarify the distinction between SOGs and SVGs, and to provide additional evidence supporting the biological significance of the identified SOGs. Our response is divided into three key points:

(1) Clarification of the conceptual differences between SOGs and SVGs

To better illustrate the fundamental difference between SOGs and SVGs, we designed a toy model to simulate spatial transcriptomics data, whose gene expression patterns are shown in Fig. S14. In this model, the first four genes directly determine the spatial coordinates of the cells, representing genes that are truly associated with spatial organization. The remaining six genes are generated based on a spatial weight matrix, leading to high spatial autocorrelation but no direct relationship with spatial location, representing SVGs in a computational sense (Details in lines 629-646). When applying iSORT, the model correctly identifies the first four genes as top SOGs (Supplementary Table 2). In contrast, traditional spatial variability metrics such as Moran's I rank these genes lower, giving priority to genes with high spatial autocorrelation but no role in spatial organization (Supplementary Table 3). This clearly demonstrates that the SOGs identified by iSORT actively influence tissue structure, whereas SVGs reflect spatial expression variability without necessarily impacting spatial organization.

The experiment has been added to the revised manuscript in the section "SOG analysis and identification of atherosclerosis-related biomarkers in human artery experiments," specifically in the paragraph "SOGs in a simulated experiment" (Line 306-324).

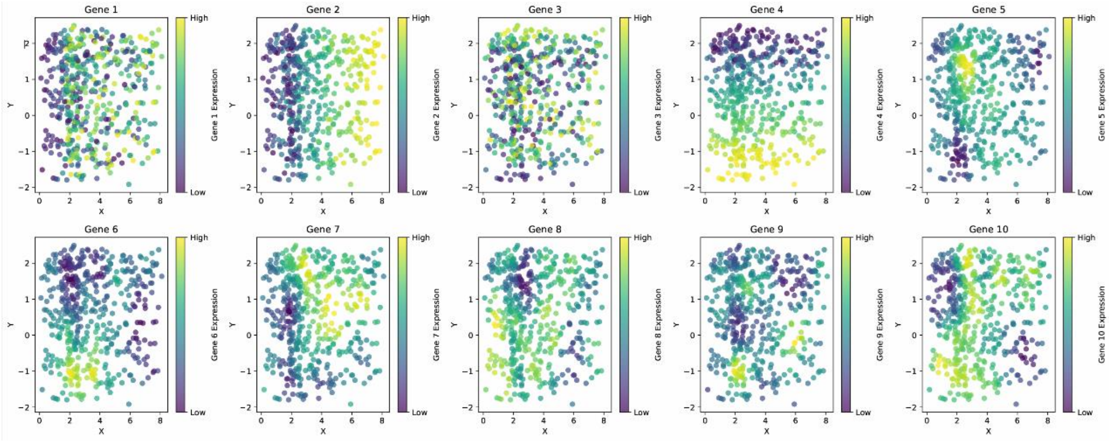

Supplementary Figure 14. Spatial distribution of the 10 genes in the toy model simulation. The plots show how each gene's expression varies across the simulated spatial coordinates.

Gene Index	1	2	3	4	5	6	7	8	9	10
SOG Score	0.6447	2.1179	0.2332	0.8623	0.1360	0.0870	0.1006	0.1053	0.0928	0.1049

Supplementary Table 2: iSORT's SOG scores for the genes

Gene Index	1	2	3	4	5	6	7	8	9	10
Moran's I	0.2751	0.8831	0.1095	0.9200	0.9708	0.9801	0.9440	0.9166	0.8840	0.9582

Supplementary Table 3: Moran's I values for the genes

Please find the updated version of the paragraph below:

(Line 306–324) SOGs in a simulated experiment.

To distinguish between SOGs and SVGs, we used a ST toy model, as described in the Methods section. In this model, we generated a gene expression matrix and spatial coordinates, incorporating spatial dependencies to simulate realistic gene distributions. The first four genes were used to determine the spatial coordinates, while the remaining six genes were generated based on spatial weights with high spatial autocorrelation. Based on Moran's I index, the spatial autocorrelation of the first four genes was relatively low, placing them behind the other six genes in terms of spatial variability (Supplementary Table 2). In contrast, the remaining six genes exhibited high spatial autocorrelation, which was visually apparent as a spatial clustering effect Fig. S14). As a result, these six genes were identified as

SVGs. However, in this model, the spatial positions were solely determined by the first four genes, as they directly influenced the tissue's spatial organization. After training iSORT, we performed SOG scoring, where iSORT successfully identified the first four genes as SOGs, ranking them as the top genes (Supplementary Table 3). This result highlights that iSORT recognizes the significant influence of these genes on the spatial structure, despite their lower spatial autocorrelation compared to the other genes. This experiment demonstrates the ability of iSORT to accurately distinguish SOGs from SVGs, providing insights into the genetic determinants of spatial organization.

(Line 629–646) Toy model of spatial transcriptomics.

To simulate spatial transcriptomics data and distinguish between SOG and SVG, we developed a toy model that generates gene expression and spatial coordinates with spatial dependencies.

The gene expression matrix $X \in R^{n\times m}$ was created, where X_{ij} represents the expression of gene j in sample i. The expression values for the first four genes were sampled from a uniform distribution $U(0, 2)$, and spatial coordinates were generated as:

$$y_{i,1} = 2 \cdot sin(X_{i,1}) + 1.5 \cdot X_{i,2}^2 + \mathcal{N}(0, R)$$

$$y_{i,2} = -exp(-X_{i,3}) + 2.5 \cdot cos(X_{i,4}) + \mathcal{N}(0, R)$$

where $\mathcal{N}(0, R)$ is Gaussian noise and R is set to 0.1.

To simulate spatially dependent genes, we introduced a spatial weight matrix based on the Euclidean distance d_{ij} between spatial coordinates, with spatial correlation controlled by parameter W:

$$w_{ij} = exp\left(-\frac{d_{ij}}{W}\right)$$

In our simulation, W was set to 0.6. Gene expression values for the remaining genes were generated as:

$$X_i = b + \sum_j w_{ij} \cdot \mathcal{N}(0,1)$$

This ensures high spatial autocorrelation and a strong Moran's I index, with the first four genes determining spatial positions and the remaining six genes exhibiting high spatial correlation.

(2) Spatial expression patterns of SOGs in real-world data (DLPFC dataset)

To provide direct evidence of the biological relevance of SOGs, we have supplemented the analysis of the DLPFC dataset with the spatial expression patterns of the top 20 SOGs. These genes are visible in both the original tissue and the reconstructed spatial organization produced by iSORT. The results show that the identified SOGs exhibit consistent and distinct spatial expression patterns, which align with the known layer-specific organization in the DLPFC. This suggests that iSORT effectively captures biologically meaningful spatial signals that are crucial for maintaining tissue structure. These visualizations and analyses have been added to the revised manuscript in Figs. S15, S16 and are discussed in lines 342-346.

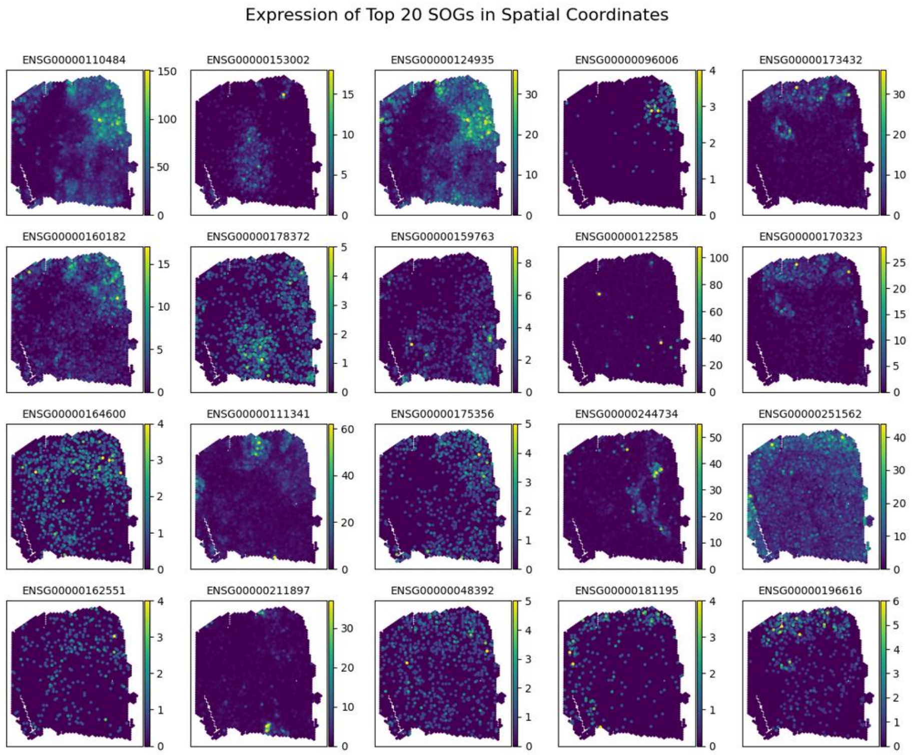

Supplementary Figure 15. Spatial expression patterns of the top 20 SOGs in the

original slice 151674. Spatial expression patterns of the top 20 SOGs in the original DLPFC tissue. These genes exhibit distinct spatial expression patterns that correspond to the known layer-specific organization within the DLPFC.

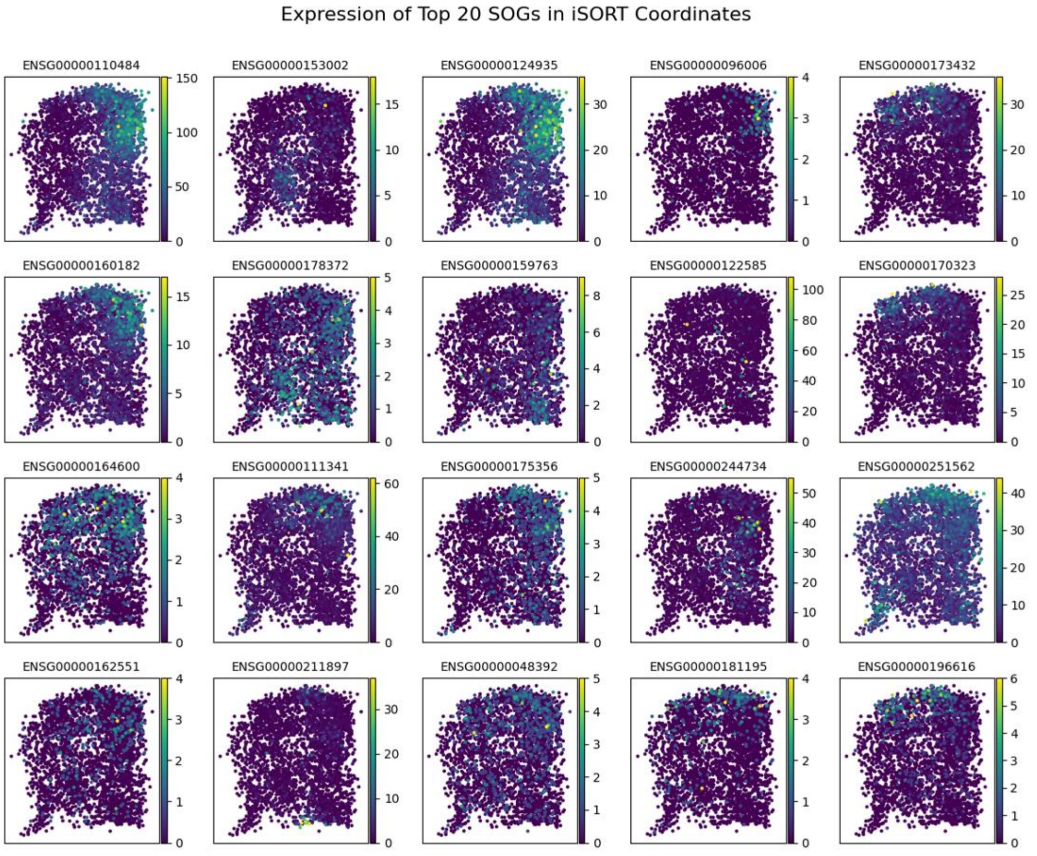

Supplementary Figure 16. Spatial expression patterns of the top 20 SOGs in the reconstructed slice 151674.

<u>**Please find the updated version of the paragraph below**</u>**:**

> **(Line 342–346) Additionally, we visualized the expression patterns of the top 20 SOGs in both the original ST and the reconstructed space (Figs. S15 and S 16). These visualizations show how the expression of these key genes correlates with the spatial structure, further supporting their critical role in maintaining the tissue's spatial organization.**

(3) Biological significance and disease relevance of SOGs (arterial data analysis)

In our manuscript, we collaborated with the Department of Cardiology at

Zhongshan Hospital, Fudan University, Shanghai, to apply iSORT to arterial tissue samples from healthy individuals and patients with atherosclerosis. Comparative analysis of the SOGs between these two conditions revealed that the identified genes are closely associated with disease progression and reflect significant spatial structural changes in the diseased tissues. This strongly supports the biological relevance of SOGs in a pathological context and highlights their potential role in understanding spatially organized diseases. Additionally, we performed Gene Ontology (GO) enrichment analysis on the identified SOGs, as shown in the Supplementary Note 8, Figs. S20 and S21. The results confirmed that these genes are involved in biological processes related to tissue structure and disease development. To better explain this point, we have revised the section 'SOG analysis and identification of atherosclerosis-related biomarkers in human artery experiments,' specifically the 'Human artery sequencing and analysis of atherosclerosis-related SOGs' subsection (line 374-376, 390-393), to provide a clearer and more detailed description of these findings.

Please find the updated version of the paragraph below:

(Line 374–393) We found 16 overlapping SOGs which are not only closely associated with vascular function but also regulate vascular spatial structure. Among them, *TNN* achieved the highest I_g score, which plays a crucial role in facilitating integrin binding and is essential for cell adhesion, migration, and proliferation. *TNN* is also important in neuronal generation and osteoblast differentiation, further underscoring its significance in vascular structure and function. Meanwhile, a number of genes found by I_g, such as *RFLNA*, *SPN*, and *EZH2*, are proved to be associated with AS. We further performed the GO enrichment analysis on the top-50 SOGs . The GO analysis revealed that 11 out of the top 20 results in GO Biological Processes (BP) were common to both the normal and AS samples (Supplementary Table 9, Supplementary Table 10, Fig. S20 and Fig. S21). The shared BP terms indicated commonality in core biological

functions, while the unique BP terms appeared in the AS sample were highly related to the abnormal vascular mineralization during AS process. More detailed analysis of the AS related pathology can be found in Supplementary Note 8. These results indicate that the SOGs identified by iSORT contributed to maintaining vascular function and could serve as biomarkers for the study of AS. Further gene and functional analysis suggests that these SOGs regulate vascular spatial structure and are involved in the pathological processes of the vessel, underscoring their important role in spatial tissue reconstruction.

We sincerely appreciate your feedback, which has helped us strengthen the clarity and biological interpretation of our findings.

5. The results in Fig. 5 is confusing. It employs UMAP reduction to present the pseudo-time and RNA velocity results of scTour, while iSORT's results are shown in physical space. It might be better to use a consistent format to demonstrate and compare the two different methods.

Reply 2.5: We sincerely appreciate your feedback and understand your concerns regarding the differences in visualization formats between scTour and iSORT.

In response to your comments, we clarified the rationale behind using different visualization formats in the manuscript on page 12 (line 401-408). This choice was deliberate, aiming to highlight a key advantage of iSORT. Specifically, iSORT projects the RNA velocity inferred by scTour back into the physical spatial organization, allowing us to observe how cellular developmental trajectories unfold within actual tissue structures. The purpose of this projection is to explore whether mapping RNA velocity to physical space can reveal spatial patterns and biological insights that might be obscured or distorted in UMAP-reduced visualization. The results show that iSORT captures more detailed and biologically relevant spatial dynamics, such as the clear progression from white matter (WM) to layer 1, which scTour fails to represent

accurately in the reduced space. This comparison emphasizes the unique value of analyzing RNA velocity in real physical space.

Furthermore, we added a new paragraph, "SpaRNA velocity on mouse embryo dataset," to the section " SpaRNA velocity and pseudo-growth trajectories on human DLPFC, developmental heart and mouse embryo dataset" on page 12 (line 425-439). In this paragraph, we presented the application of iSORT on mouse embryo dataset. iSORT successfully predicted spatial differentiation trajectories of distinct cell types, revealing the spatial developmental patterns (see Fig. 6(i) in the main text and Fig. S23). For instance, we observed anterior-dorsal differentiation of gut tube cells and differentiation of mesodermal cells towards splanchnic mesoderm, while mesodermal and ectodermal cells followed distinct differentiation trajectories with clear spatial localization patterns. These findings align with known biological processes of embryonic development.

Additionally, in the discussion, we explored SIRV, which integrates scRNA-seq and ST to infer RNA velocity in physical space, on the same dataset (line 496-512). It showed partial consistency with iSORT in the differentiation trajectories of certain cell types (Fig. S23). However, unlike SIRV, iSORT does not require additional scRNA-seq data when inferring SpaRNA velocity. These findings further <u>highlight the effectiveness of iSORT in capturing spatial developmental trends</u>.

<u>Please find the updated version of the paragraph below</u>:

(Line 401-408) Since scTour is a method that infers pseudo-time and RNA velocity based solely on gene expression, which is not spatially resolved, its results are typically visualized in reduced-dimensional space. In this case, the pseudo-time and RNA velocity inferred by scTour in the reduced two-dimensional space could not capture the correct growth of Layer 1 (Figs. 6(b) and (c)). Pseudo-time inferred from the gene expression space exhibited discontinuities on the ST slice (Fig. 6(d)), such as between Layer 2 and Layer 3 (Fig. 6(e)).

(Line 425-439) To further assess iSORT's ability to infer spatial RNA velocity, we tested its performance on a more complex mouse embryo tissue dataset, which was previously used for spatial reconstruction in our study. The data contains rich cellular diversity across spatially resolved samples, which provided an excellent basis for evaluating spatial velocity inference. Unlike other methods for inferring RNA velocity, which require paired scRNA-seq data to estimate spliced and unspliced gene expressions, iSORT infers spatial RNA velocity directly from ST data.

In this analysis, iSORT successfully predicted the spatial differentiation trajectories of different cell types, revealing distinct patterns of spatial development (Figs. 6(i) and Fig. S23). We observed the anterodorsal differentiation of gut tube cells and the differentiation of mesodermal cells toward splanchnic mesoderm. Meanwhile, mesodermal and ectodermal cells followed distinct differentiation trajectories with clear spatial localization patterns, which are consistent with known biological processes of embryonic development.

(Line 496-512) Moreover, iSORT's mapping-based approach allows it to incorporate the concept of RNA velocity to infer pseudo-growth trajectories on ST data, thereby capturing dynamic changes within tissues. By inferring SpaRNA velocity, iSORT not only reflects the organizational and differentiation processes within tissues, but also reveals spatiotemporal dynamics. More recently, SIRV was developed to infer RNA velocity in the physical space of ST by integrating scRNA-seq and ST data. Specifically, it utilizes splicing information from scRNA-seq to predict spliced and unspliced RNA levels in the ST data, enabling RNA velocity inference within the tissue structure. In contrast, we propose a quantity named SpaRNA velocity. Instead of relying on external sequencing references, SpaRNA velocity is computed solely from ST data and projected onto the ST slice, providing pseudo-growth trajectories that model how cells transition from their progenitors in space. Other methods of RNA velocity, such as scVelo, can also be appropriately

modified for SpaRNA velocity inference. Besides, the inference of pseudo-time is another important aspect in assessing cell developmental status. Inference of SpaRNA velocity directly from ST data provides a promising framework for studying pseudo-time and could serve as a meaningful topic for future research.

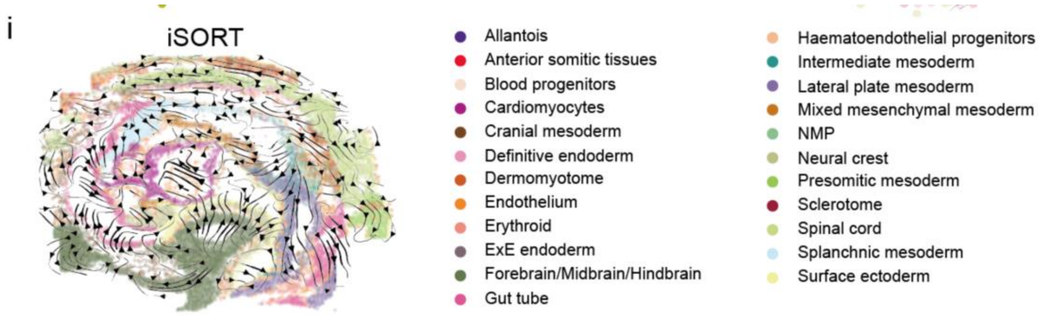

Figure 6. (i) SpaRNA velocity inferred by iSORT on the mouse embryo dataset. iSORT successfully predicted the spatial differentiation trajectories of different cell types, revealing the patterns of spatial development.

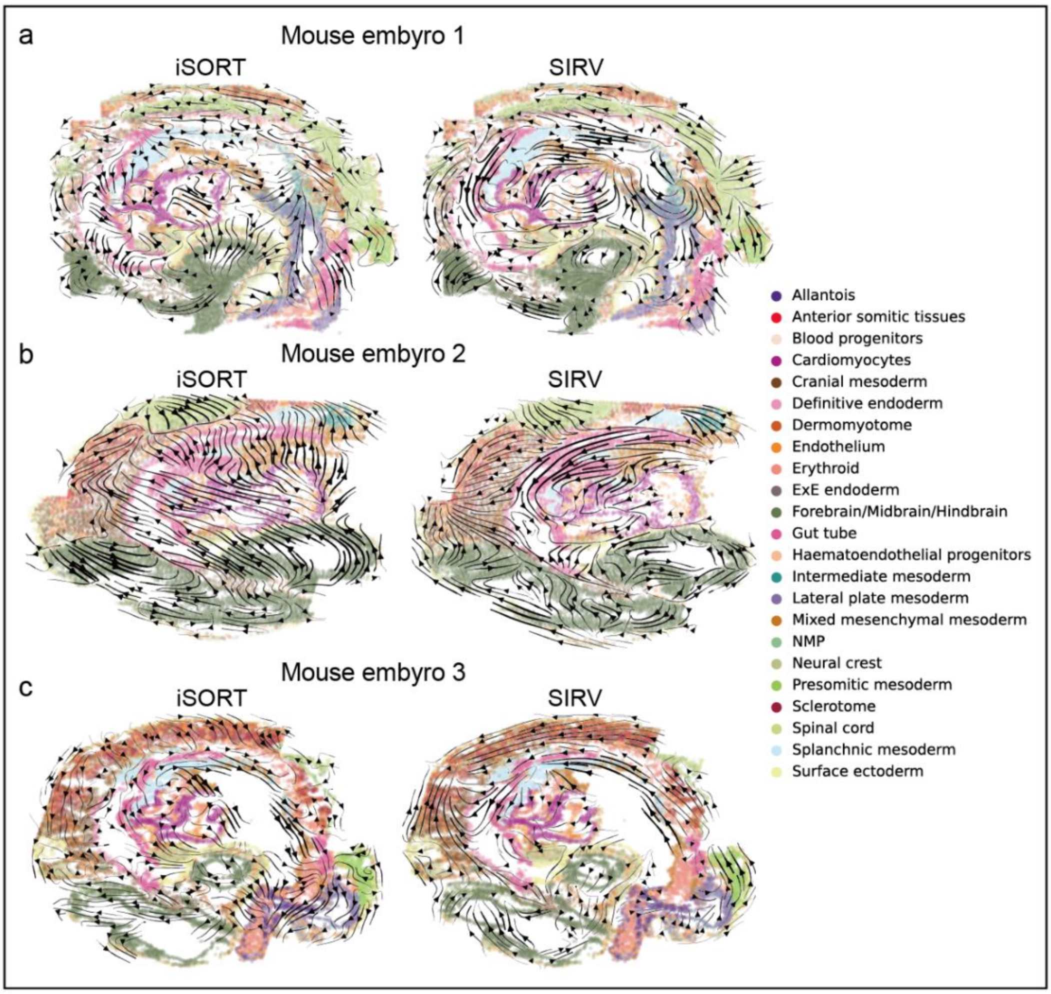

Supplementary Figure 23. Comparison of SpaRNA velocity inferred by iSORT and SIRV on the mouse embryo dataset. Both methods successfully predicted the spatial differentiation trajectories of different cell types, revealing similar patterns of spatial development.

Response to Reviewer #3

Reviewer #3:

Remarks to the Author:

In this manuscript, the authors established iSORT, a novel mapping method between single-cell and spatial transcriptomes. They also proposed two downstream analysis methods for spatial RNA velocity estimation and spatial gene identification, respectively. I have the following concerns:

Reply 3.0: We appreciate your thoughtful summary and encouraging feedback on our study. We have carefully revised the manuscript to resolve all the concerns raised. Our point-by-point responses are provided below.

1. iSORT is a reference-based mapping method. The authors should compare iSORT with state-of-the-art reference-based mapping methods such as CytoSPACE (https://www.nature.com/articles/s41587-023-01697-9), STEM (https://www.nature.com/articles/s42003-023-05640-1) and Celloc (https://onlinelibrary.wiley.com/doi/10.1002/smsc.202400139).

Reply 3.1: We sincerely appreciate your valuable feedback and for highlighting the necessity of comparing iSORT with the latest reference-based mapping methods. In response to your suggestion, we have conducted a comprehensive benchmarking analysis, directly comparing iSORT with state-of-the-art methods such as CytoSPACE and Celloc. The results demonstrate that iSORT consistently achieves superior or comparable performance in terms of both accuracy and computational efficiency. These findings have been incorporated into Section "Benchmarking iSORT in reconstructing the spatial organization of single cells" and are clearly presented in Figs. 2(a), 2(b) and Fig. S1.

Regarding STEM, we fully acknowledge its relevance as a leading reference-based mapping method. However, due to its extremely **high memory consumption**, we were unable to run STEM on the DLPFC dataset. Despite the dataset's relatively small size,

it exceeded our available GPU memory resources, **surpassing 80 GB of GPU memory.** Specifically, STEM requires a substantial amount of GPU memory, even for small datasets, which presents significant limitations for practical use. Notably, the authors of STEM have also acknowledged this issue on their GitHub repository (https://github.com/WhirlFirst/STEM/issues/3), stating: "STEM does require a high CUDA memory for optimizing". This limitation highlights the practical challenges of applying STEM to real-world datasets, even those of modest size.

In addition to addressing your specific suggestion, we have further expanded our benchmarking analysis to include comparisons with widely used spot deconvolution algorithms such as RCTD, CARD and Redeconve. Specifically, in the main text on pages 4-5 (line 117-145), we expanded the benchmark to include comparisons with 10 methods to comprehensively evaluate the performance of iSORT. These methods encompass mainstream techniques based on mapping and deconvolution, covering most of the major approaches in the field. The results demonstrate that iSORT excels in accurately reconstructing the spatial organization of single cells and is also competitive compared to deconvolution-based methods (as shown in Fig. 2(a) of the main text). Additionally, we applied the previously used quantitative metrics to newly included methods, demonstrating that iSORT outperforms the other 10 methods across all metrics (see Fig. 2(b) in the main text and Fig. S1).

We have also included references to relevant studies in this section, as follows:

[43] Vahid MR, Brown EL, Steen CB, Zhang W, Jeon HS, Kang M, et al. High-resolution alignment of single-cell and spatial transcriptomes with CytoSPACE. Nat Biotechnol. 2023;41(11):1543–1548.

[44] Yin W, Wu X, Chen L, Wan Y, Zhou Y. Accurate and Flexible Single Cell to Spatial Transcriptome Mapping with Celloc. Small Science. 2024;4(10):2400139.

We sincerely thank you for your constructive feedback, which has significantly strengthened the comprehensiveness and robustness of our performance evaluation.

Please find the updated version of the paragraph below:

(Line 117-145) The output of iSORT were compared with the ground truth (ST slice ID151674) as well as five other existing methods capable of predicting single-cell spatial positions: scSpace, Tangram, novoSpaRc, CeLEry, and CellTrek. Additionally, we performed a comparison with five spot deconvolution methods: RCTD, CARD, Redeconve, CytoSPACE, and Celloc, to provide a more comprehensive benchmarking analysis (Fig. 2(a)). It is worth noting that Tangram is designed for spot deconvolution, while novoSpaRc is designed for imputing undetected genes, but both methods use probabilistic models and can be used for position reconstruction. Detailed information on the other methods is shown in Supplementary Note 2. In the group of methods for spatial position reconstruction, iSORT, Tangram, novoSpaRc, and CeLEry could reconstruct the shape of ST slice ID151674 from gene expressions, while iSORT, scSpace, novoSpaRc, and CeLEry could distinguish different layers with clear boundaries. For the spot deconvolution methods, we selected the cell type with the highest proportion in each spot as the dominant cell type for that spot, providing a unified basis for comparison. Each method was able to roughly distinguish the different layers, particularly the white matter (WM) layer. Among the spot deconvolution methods, Redeconve and RCTD showed the clearest distinction of layers. In contrast, CARD did not predict the presence of Layer 6 cell types, as no spot in the CARD predictions had Layer 6 as the most abundant cell type. Celloc and CytoSPACE showed distinct results for layers other than the WM layer, but the overall layer differentiation was less pronounced compared to Redeconve and RCTD. Four different indicators were used to evaluate the performance of the eleven methods (Fig. 2(b)), i.e. the intra-layer similarity S_C, the normalized density distribution ρ_C, the aggregative volume index A_C, and the aggregative perimeter index P_C (Definitions and details are provided in Supplementary Note 5). Values of the indicators measure the performance of algorithms in reconstructing layer structures. iSORT got an average S_C as 0.94, ρ_C as 0.84, A_C as 0.88, and P_C as 0.84, which were larger than the values obtained from the other ten methods (Fig.

2(b) and Fig. S1).

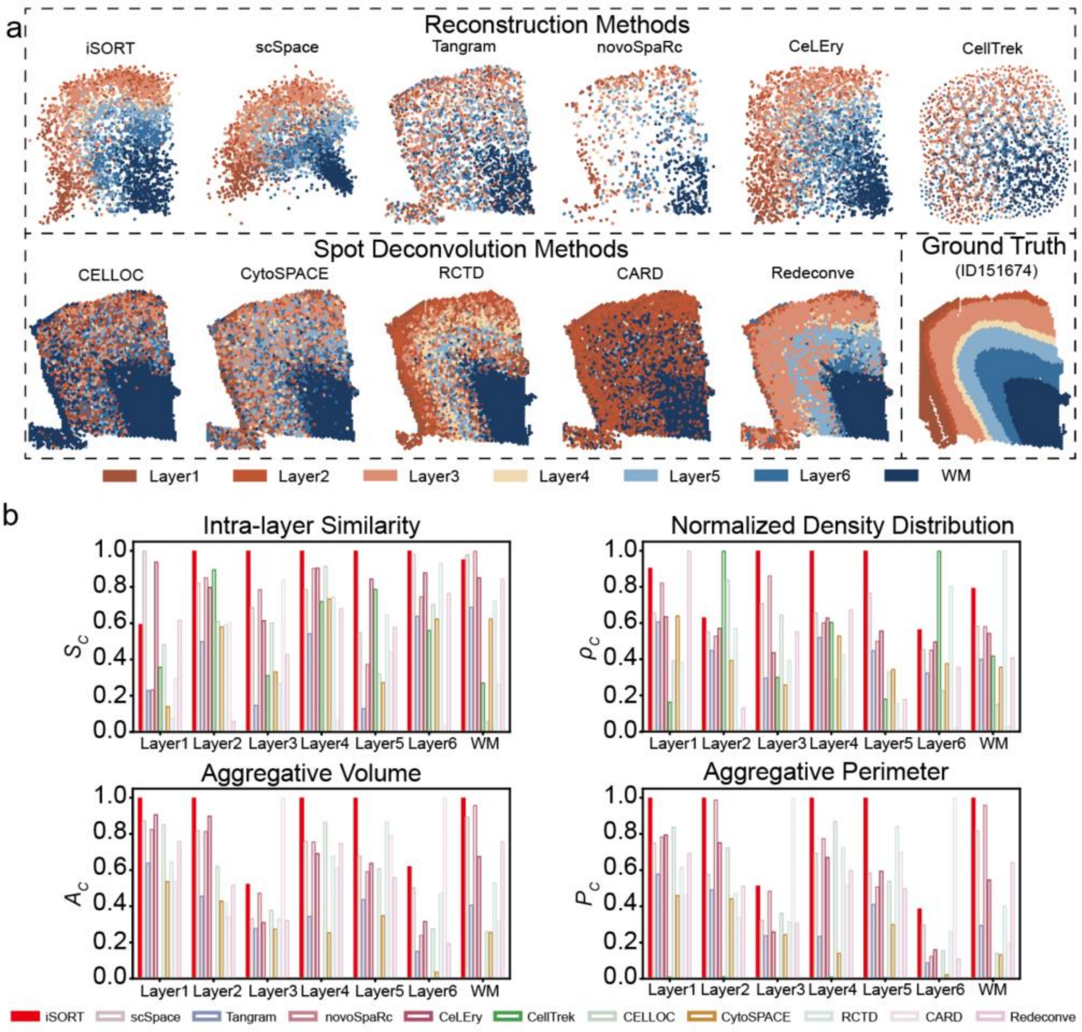

Figure 2(a) and (b). (a) Ground truth and spatial reconstruction results for ID151674 in DLPFC dataset by eleven algorithms: iSORT, scSpace, Tangram, novoSpaRc, CeLEry, CellTrek, RCTD, CARD, Redeconve, CytoSPACE, and Celloc, where Tangram, RCTD, CARD, Redeconve, CytoSPACE, and Celloc are designed for spot deconvolution, whereas novoSpaRc is designed for imputing the undetected genes. Different colors represent different cortical regions. (b) Bar charts to compare the performance of eleven algorithms based on four indicators: intra-layer similarity S_C, normalized density distribution ρ_C, aggregative volume index A_C and aggregative perimeter index P_C of each layer. The indicators of iSORT (the red filled bars) are above 0.875 in average, larger than the other ten algorithms.

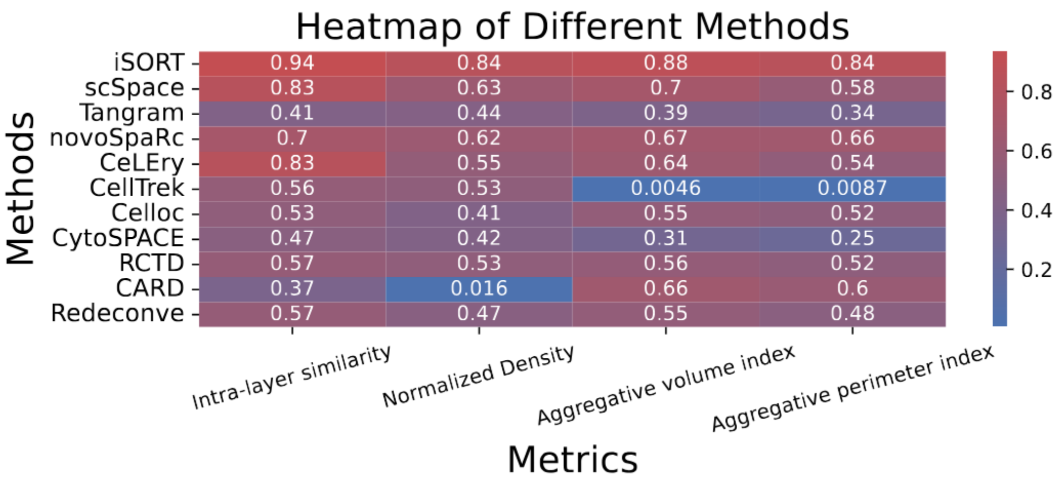

Supplementary Figure 1. Heatmap of four clustering metrics. Heatmap of four metrics (Mean of intra-layer similarity, mean of normalized density, mean of aggregative volume index and mean of aggregative perimeter index) of different methods (iSORT, scSpace, Tangram, novoSpaRc, CeLEry, CellTrek, RCTD, CARD, Redeconve, CytoSPACE, and Celloc). The redder the colour, the better the clustering of the hierarchies. iSORT outperforms other methods in layering performance in an average sense.

2. Inclusion of multiple ST references is a highlight feature of iSORT, but the tests demonstrating such advantage are not sufficient.

Reply 3.2: Thank you very much for your thoughtful feedback. We appreciate your recognition of iSORT's ability to integrate multiple spatial transcriptomics (ST) references. We understand your concerns and would like to clarify that we have conducted comprehensive experiments to demonstrate this advantage:

(1) Comprehensive comparison between single-slice and multi-slice integration

We conducted a systematic comparison between single-slice and multi-slice integration in iSORT. Specifically, we evaluated how multi-slice integration improves spatial reconstruction accuracy compared to using only a single ST reference. The results clearly show that integrating multiple ST references leads to better alignment and more accurate single-cell spatial positioning.

(2) Application to artery data from different biological states

We further validated the effectiveness of multi-slice integration using artery tissue datasets from different biological states. By integrating slices from different conditions, iSORT was able to identify key spatial-organizing genes (SOGs) that change with biological states, providing deeper insights into the mechanisms behind spatial organization.

To emphasize these points, we have added a section in the Discussion highlighting the advantages of using iSORT with multi-slice data (page14, line 484-495). This addition underscores how iSORT can leverage multi-slice information to enhance spatial reconstruction and provide more comprehensive insights into tissue organization.

Once again, thank you for your valuable feedback, which has allowed us to further clarify and highlight the advantages of iSORT.

Please find the updated version of the paragraph below:

(Line 484-495) Traditional spot deconvolution methods typically assign single cells to spots or integrate data to infer the cell type proportions within spots. Although these methods achieve high accuracy, they are limited to analyzing a single ST slice and cannot utilize information from multiple slices. iSORT's multi-slice training approach overcomes this limitation. One respect is that integrating data from multiple slices significantly improves the accuracy of spatial reconstruction. Another is that incorporating slices in different states enables iSORT effectively identify key genes that determine spatial structures. This capability makes iSORT uniquely advantageous in disease research — leveraging spatial information from multiple ST slices to identify SOGs, thus providing a method for detecting pathogenic genes. Besides the atherosclerosis described in this study, iSORT could offer a new perspective for revealing broader disease-related pathways.

3. Although the authors emphasize the introduction of transfer learning in their algorithm, I could not exactly find which part of the algorithm adopts such a transfer learning strategy in Figure 1. It seems that the term "density ratio transfer" would more accurately describe the strategy.

Reply 3.3: Thank you very much for your insightful comments. We agree that "Density Ratio Transfer" is a more accurate term to describe the strategy employed in our algorithm. In fact, density ratio transfer is a specific method within the broader field of transfer learning (Zhuang et al., 2020), particularly effective in addressing distributional shifts between datasets. Our approach leverages this concept to adjust distribution differences, enabling more efficient integration of single-cell and spatial transcriptomics data. In response to the reviewer's comments, we revised the sentence "Estimation of the density ratio is the core technique." on page 3, line 72-74 of the main text to "Estimation of the density ratio is the core technique, which is a specialized method used in transfer learning to address distributional discrepancies between domains." This revision clarifies the relationship between density ratio transfer and transfer learning more effectively.

4. For spatial RNA velocity, the authors could compare their method with the latest method SIRV (https://academic.oup.com/nargab/article/6/3/lqae100/7728020).

Reply 3.4: We sincerely appreciate the reviewer's valuable suggestions and the recommendation to compare iSORT with the latest spatial RNA velocity method, SIRV. In response to your comments, we added a subsection titled "SpaRNA velocity on the mouse embryo dataset" to the section "SpaRNA velocity and pseudo-growth trajectories in the human DLPFC, developmental heart, and mouse embryo datasets" (page 12, line 425–439) in the manuscript. Additionally, we included a discussion of the SIRV results in the Discussion section and provided the comparison results in the supplementary information (SI) (pages 14, line 496-512).

Specifically, both methods successfully predicted the spatial differentiation trajectories of distinct cell types, revealing similar spatial developmental patterns (see Fig. 6(i) in the main text and Fig. S23). For instance, we observed anterior-dorsal differentiation of gut tube cells and differentiation of mesodermal cells towards visceral mesoderm. Meanwhile, mesoderm and ectoderm cells followed distinct differentiation trajectories with clear spatial localization patterns, consistent with the known biological processes of embryonic development. However, unlike SIRV, iSORT does not rely on additional scRNA-seq data when inferring SpaRNA velocity. This comparison further highlights the competitiveness and unique advantages of iSORT in accurately capturing spatial RNA velocity patterns and developmental trajectories.

We have also included reference to relevant studies as follows:

[71] Abdelaal T, Grossouw LM, Pasterkamp RJ, Lelieveldt BP, Reinders MJ, Mahfouz A. SIRV: Spatial inference of RNA velocity at the single-cell resolution. NAR genom bioinform. 2024;6(3):lqae100.

We sincerely thank you for this insightful suggestion, which has helped us further strengthen the validation of our method.

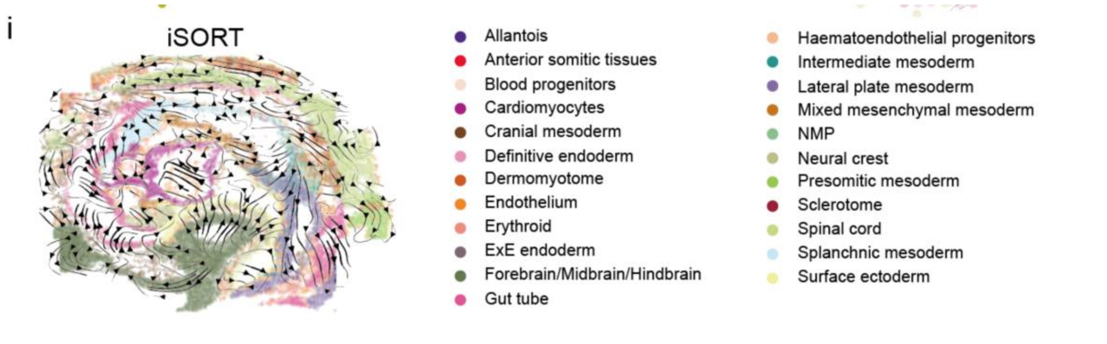

Figure 6. (i) SpaRNA velocity inferred by iSORT on the mouse embryo dataset. iSORT successfully predicted the spatial differentiation trajectories of different cell types, revealing the patterns of spatial development.

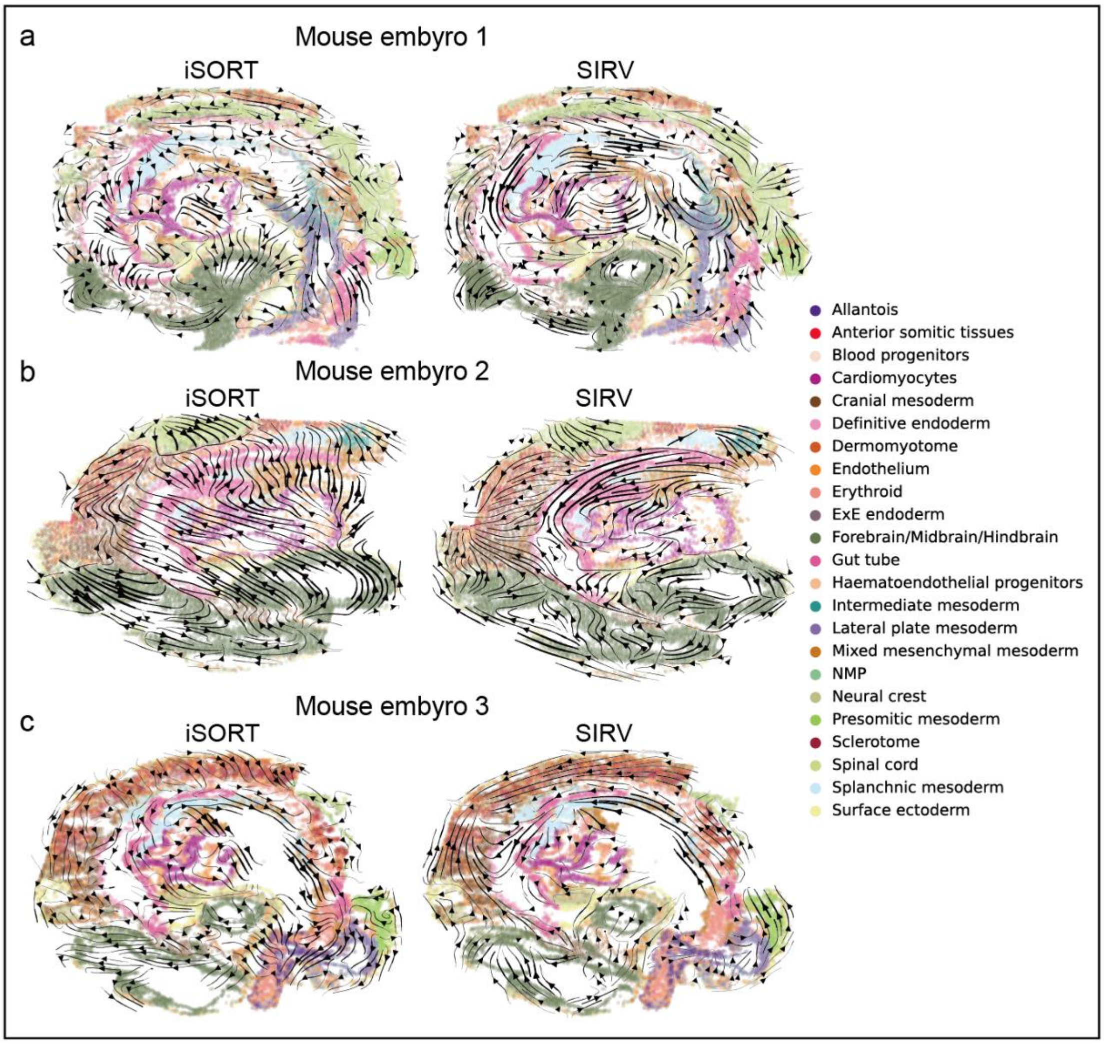

Supplementary Figure 23. Comparison of SpaRNA velocity inferred by iSORT and SIRV on the mouse embryo dataset. Both methods successfully predicted the spatial differentiation trajectories of different cell types, revealing similar patterns of spatial development.

<u>**Please find the updated version of the paragraph below**</u>:

(Line 425-439) To further assess iSORT's ability to infer spatial RNA velocity, we tested its performance on a more complex mouse embryo tissue dataset, which was previously used for spatial reconstruction in our study. The data contains rich cellular diversity across spatially resolved samples, which provided an excellent basis for evaluating spatial velocity inference. Unlike other methods for inferring RNA velocity, which require paired scRNA-seq data to estimate spliced and

unspliced gene expressions, iSORT infers spatial RNA velocity directly from ST data.

In this analysis, iSORT successfully predicted the spatial differentiation trajectories of different cell types, revealing distinct patterns of spatial development (Figs. 6(i) and Fig. S23). We observed the anterodorsal differentiation of gut tube cells and the differentiation of mesodermal cells toward splanchnic mesoderm. Meanwhile, mesodermal and ectodermal cells followed distinct differentiation trajectories with clear spatial localization patterns, which are consistent with known biological processes of embryonic development.

(Line 496-512) Moreover, iSORT's mapping-based approach allows it to incorporate the concept of RNA velocity to infer pseudo-growth trajectories on ST data, thereby capturing dynamic changes within tissues. By inferring SpaRNA velocity, iSORT not only reflects the organizational and differentiation processes within tissues, but also reveals spatiotemporal dynamics. More recently, SIRV was developed to infer RNA velocity in the physical space of ST by integrating scRNA-seq and ST data. Specifically, it utilizes splicing information from scRNA-seq to predict spliced and unspliced RNA levels in the ST data, enabling RNA velocity inference within the tissue structure. In contrast, we propose a quantity named SpaRNA velocity. Instead of relying on external sequencing references, SpaRNA velocity is computed solely from ST data and projected onto the ST slice, providing pseudo-growth trajectories that model how cells transition from their progenitors in space. Other methods of RNA velocity, such as scVelo, can also be appropriately modified for SpaRNA velocity inference. Besides, the inference of pseudo-time is another important aspect in assessing cell developmental status. Inference of SpaRNA velocity directly from ST data provides a promising framework for studying pseudo-time and could serve as a meaningful topic for future research.

5. For spatial gene identification, the rationale for equation (13) is not clear. In addition,

the authors could perform ssGSEA/GSVA analysis to check whether the spatial-organizing genes found by their method are more enriched for known key development-related genes or disease genes.

Reply 3.5: We thank the reviewer for the valuable comments.

Regarding Equation (13) (i.e., $||\partial_x \boldsymbol{f}(\boldsymbol{x})||$), our assumption is that spatial positions are related to gene expression through a functional relationship $\boldsymbol{f}$, i.e., $\boldsymbol{f}(\boldsymbol{x}) = \boldsymbol{y}$, where $\boldsymbol{x}$ and $\boldsymbol{y}$ represent gene expression and spatial positions, respectively, and $\boldsymbol{f}$ can be learned through iSORT. Therefore, calculating the gradient of $\boldsymbol{f}$ with respect to $\boldsymbol{x}$ represents how small changes in gene expression lead to changes in spatial position. If the gradient is large, it indicates that changes in gene expression have a significant impact on spatial position, meaning the gene has a high importance in determining spatial location; if the gradient is small, it suggests that gene expression has a lesser effect on spatial position. By calculating the expectation of the gradient across all cells, we obtain the average impact of the gene on the entire tissue, taking into account its effect across different cells. This allows us to comprehensively assess the gene's contribution to spatial organization, avoiding biases from local effects. This approach, which calculates the absolute value of the gradient of gene expression on spatial position, is similar to the saliency mapping method used in computer vision. Saliency mapping aims to identify which features (such as image pixels) have the most significant impact on the model's output by calculating the gradient or other influence metrics of the input features.

Regarding ssGSEA/GSVA, we would like to clarify its applicability. The primary goal of identifying SOGs in our study is to capture genes that are critical for establishing and maintaining spatial organization, with SOGs emphasizing their role in the spatial structure of tissues. Since ssGSEA/GSVA is designed to assess the enrichment of predefined gene sets across different samples, rather than evaluating spatial organization, it may not be directly applicable for assessing SOGs. To validate the biological significance of SOGs, we conducted the following experiments to better

assess the spatial key role of SOGs and their potential as key disease-related genes:

(1) In-silico SOG knockout experiment: In the DLPFC experiment, we investigated the impact of SOG knockout on spatial reconstruction to examine the role of SOGs in the formation of spatial organization.

(2) Spatial expression patterns of SOGs: In the DLPFC experiment, we supplemented the spatial expression patterns of top-ranking SOGs, revealing meaningful spatial expression consistent with tissue structure. (Line 342-346)

(3) Gene Ontology (GO) enrichment analysis: In the artery reconstruction experiment, we performed GO enrichment analysis on top-ranking SOGs, and the results showed significant enrichment in biological processes related to tissue development and spatial organization.

Furthermore, in collaboration with the Department of Cardiology at Zhongshan Hospital, Fudan University, we analyzed arterial tissue samples from healthy individuals and atherosclerosis patients. The identified SOGs highlighted key spatial genes associated with disease progression and tissue remodeling, supporting the biological relevance of SOGs in pathological contexts.

We sincerely appreciate the reviewer's suggestions, which have allowed us to further clarify the unique role of SOGs and provide additional analyses to strengthen the validation of our findings.

Please find the updated version of the paragraph below:

(Line 342-346) Additionally, we visualized the expression patterns of the top 20 SOGs in both the original ST and the reconstructed space (Figs. S15 and S16). These visualizations show how the expression of these key genes correlates with the spatial structure, further supporting their critical role in maintaining the tissue's spatial organization.

6. To better quantify the performance, the authors could compare their spatial-organizing gene identification method with the previous methods in simulated datasets,

using a benchmarking strategy similar to the recent report (https://genomebiology.biomedcentral.com/articles/10.1186/s13059-023-03145-y).

Reply 3.6: We thank the reviewer for the valuable suggestions. We appreciate your recommendation to benchmark our SOG identification method against existing methods, similar to the strategy used in the reference study. However, we would like to clarify that the concept of SOG in iSORT is different from the commonly used concept of SVGs. While SVGs primarily capture genes with spatially heterogeneous expression patterns (often measured by spatial autocorrelation metrics such as Moran's I), SOGs in iSORT specifically refer to genes that actively determine and influence spatial organization and tissue structure. This distinction makes it less appropriate to directly benchmark SVG detection methods, as these methods are designed to identify different types of spatial features.

To better illustrate the difference between SOGs and SVGs, we designed a toy model simulating spatial transcriptomics data. In this model, the first four genes are explicitly defined by equations to directly determine spatial positions, while other genes exhibit high spatial autocorrelation but do not participate in spatial positioning, representing SVGs (The spatial distribution is shown in Fig. S14 and model details are in line 629-646). In our results, iSORT correctly identifies the true spatial-organizing genes, while traditional spatial variability metrics such as Moran's I prioritize genes with high spatial autocorrelation, further emphasizing the conceptual and functional differences between SOGs and SVGs.

To further clarify this, this experiment has been added to the revised manuscript in the section “SOG analysis and identification of atherosclerosis-related biomarkers in human artery experiments,” specifically in the paragraph “SOGs in a simulated experiment” (Line 306-324).

We have also included reference to relevant studies as follows:

[32] Chen C, Kim HJ, Yang P. Evaluating spatially variable gene detection methods for spatial transcriptomics data. Genome Biol. 2024;25(1):18.

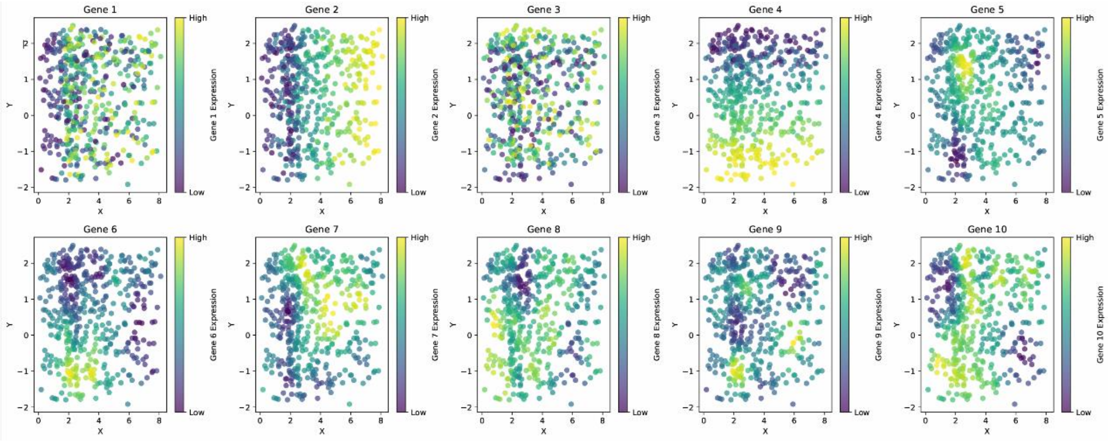

Supplementary Figure 14. Spatial distribution of the 10 genes in the toy model simulation. The plots show how each gene's expression varies across the simulated spatial coordinates.

Gene Index	1	2	3	4	5	6	7	8	9	10
SOG Score	0.6447	2.1179	0.2332	0.8623	0.1360	0.0870	0.1006	0.1053	0.0928	0.1049

Supplementary Table 2: iSORT's SOG scores for the genes

Gene Index	1	2	3	4	5	6	7	8	9	10
Moran's I	0.2751	0.8831	0.1095	0.9200	0.9708	0.9801	0.9440	0.9166	0.8840	0.9582

Supplementary Table 3: Moran's I values for the genes

Please find the updated version of the paragraph below:

(Line 306-324) SOGs in a simulated experiment. To distinguish between SOGs and SVGs, we used a toy model of ST, as described in the Methods section. In this model, we generated a gene expression matrix and spatial coordinates, incorporating spatial dependencies to simulate realistic gene distributions. The first four genes were used to determine the spatial coordinates, while the remaining six genes were generated based on spatial weights with high spatial autocorrelation. Based on Moran's I index, the spatial autocorrelation of the first four genes was relatively low, placing them behind the other six genes in terms of spatial variability (Supplementary Table 2). In contrast, the remaining six genes exhibited high spatial autocorrelation, which was visually apparent as a spatial clustering effect (Fig. S14). These genes were identified as SVGs. However, in this

model, the spatial positions were solely determined by the first four genes, as they directly influenced the tissue's spatial organization. After training iSORT, we performed SOG scoring, where iSORT successfully identified the first four genes as SOGs, ranking them as the top genes (Supplementary Table 3). This result highlights that iSORT recognizes the significant influence of these genes on the spatial structure, despite their lower spatial autocorrelation compared to the other genes. This experiment demonstrates the ability of iSORT to accurately distinguish SOGs from SVGs, providing insights into the genetic determinants of spatial organization.

(Line 629-646)

Toy model of spatial transcriptomics.

To simulate spatial transcriptomics data and distinguish between SOG and SVG, we developed a toy model that generates gene expression and spatial coordinates with spatial dependencies.

The gene expression matrix $X \in R^{n\times m}$ was created, where X_{ij} represents the expression of gene j in sample i. The expression values for the first four genes were sampled from a uniform distribution $U(0,2)$, and spatial coordinates were generated as:

$$y_{i,1} = 2 \cdot sin(X_{i,1}) + 1.5 \cdot X_{i,2}^2 + \mathcal{N}(0,R)$$

$$y_{i,2} = -exp(-X_{i,3}) + 2.5 \cdot cos(X_{i,4}) + \mathcal{N}(0,R)$$

where $\mathcal{N}(0,R)$ is Gaussian noise and R is set to 0.1.

To simulate spatially dependent genes, we introduced a spatial weight matrix based on the Euclidean distance d_{ij} between spatial coordinates, with spatial correlation controlled by parameter W:

$$w_{ij} = exp\left(-\frac{d_{ij}}{W}\right)$$

In our simulation, W was set to 0.6. Gene expression values for the remaining

genes were generated as:

$$\boldsymbol{X_i = b + \sum_j w_{ij} \cdot \mathcal{N}(0, 1)}$$

This ensures high spatial autocorrelation and a strong Moran's I index, with the first four genes determining spatial positions and the remaining six genes exhibiting high spatial correlation.

Reference:

Zhuang, F., Qi, Z., Duan, K., Xi, D., Zhu, Y., Zhu, H., Xiong, H., & He, Q. (2020). A comprehensive survey on transfer learning. Proceedings of the IEEE, 109(1), 43-76.
